# Black market blood transfusions for Ebola: potential for increases in other infections

**DOI:** 10.3402/gha.v7.26356

**Published:** 2014-11-17

**Authors:** Morenike O. Folayan, Brandon Brown, Aminu Yakubu

**Affiliations:** 1 Institute of Public Health, Obafemi Awolowo University, Ile-Ife, Nigeria; 2 Department of Child Dental Health, Obafemi Awolowo University, Ile-Ife, Nigeria; 3 Department of Population Health & Disease Prevention, University of California, Irvine, CA, USA; 4 Department of Health Planning and Research, Federal Ministry of Health, Abuja, Nigeria

Poor health systems and structures in countries affected by Ebola virus disease (EVD) have compounded difficulties in access to hospital care for Ebola patients. With this low healthcare access, individuals may be forced to seek alternative remedies for the management of EVD. One major study suggested that the transfusion of whole blood and serum from patients who had recovered from EVD reduces the risk of EVD-related fatality (1), with additional rigorous studies underway including patients in the current epidemic in West Africa (2–4) .

In the wake of the growing epidemic, it is possible that this alternative may bring new challenges such as the emergence of a black market trading of blood of EVD survivors (5). This may include the related risk of escalating transmission of human immunodeficiency virus (HIV) and viral hepatitis infection; two potential outcomes with far-reaching hazardous consequences for the West African region. The potential for these risks was highlighted by the World Health Organization with concerns about HIV transmission (3, 6). Still, this is the one available therapy with the potential to be implemented immediately on a large scale to help address the current epidemic (7).

Concerns about transfusion-related transmission of HIV and hepatitis infection in the sub-Saharan African region are not unfounded. Afolabi et al. (8) demonstrated that 2.0, 5.9 and 1.4% of potential blood donors in Nigeria had HIV, hepatitis B virus (HBV), and HCV, respectively. This risk was further highlighted by Apata et al., who showed the 2011 prevalence of HBV infection in blood donors was 9.8% in Guinea, 7.4% in Liberia, and 11.6% in Sierra Leone (9). Similarly, the HIV prevalence in 2011 among adults was 1.4% for Guinea, 1.0% for Liberia, and 1.6% for Sierra Leone (10).

In the face of an epidemic with a high fatality rate and low likelihood for access to quality medical care (11), the prospect of stamping out any illegal trade of EVD survivor blood is low, despite expressed concerns. Ebola affected countries may see an upsurge in the prevalence of other blood-borne infections both during and following the EVD epidemic, further taxing the healthcare system. Additional support and attention are needed to curb this possibility. It is therefore important to remain educated on proper management of EVD. Blood transfusion by unqualified health care practitioners must be avoided. In addition to public education, systems need to be put in place to address myths and misconceptions about management of EVD (12).

*Morenike O. Folayan* Institute of Public Health Obafemi Awolowo University Ile-Ife, Nigeria Department of Child Dental Health Obafemi Awolowo University Ile-Ife, Nigeria *Brandon Brown* Department of Population Health & Disease Prevention University of California Irvine, CA, USA Email: brandon.brown@uci.edu *Aminu Yakubu* Department of Health Planning and Research Federal Ministry of Health Abuja, Nigeria

## References

[1] Mupapa K, Massamba M, Kibadi K, Kuvula K, Bwaka A, Kipasa M (1999). Treatment of Ebola hemorrhagic fever with blood transfusions from convalescent patients. International Scientific and Technical Committee. J Infect Dis.

[2] Roberts M Ebola serum for Africa patients within weeks says WHO. http://www.bbc.com/news/health-29707393.

[3] Morin M WHO to issue guidelines on treating Ebola with blood, plasma therapy. http://www.latimes.com/science/sciencenow/la-sci-sn-ebola-blood-20140926-story.html.

[4] News24Nigeria Doctor gives blood for Ebola-infected Dallas nurse. http://www.news24.com.ng/World/News/Doctor-gives-blood-for-Ebola-infected-Dallas-nurse-20141014-2.

[5] World Health Organization Press conference on support to Ebola affected countries. http://www.who.int/mediacentre/multimedia/ebola_cuba_minister_presser_12SEP2014.pdf.

[6] World Health Organization Experimental therapies: growing interest in the use of whole blood or plasma from recovered Ebola patients (convalescent therapies). http://www.who.int/mediacentre/news/ebola/26-september-2014/en/.

[7] Butler D Blood transfusion named as priority treatment for Ebola. http://www.nature.com/news/blood-transfusion-named-as-priority-treatment-for-ebola-1.15854.

[8] Afolabi AY, Abraham A, Oladipo EK, Adefolarin AO, Fagbami AH (2013). Transfusion transmissible viral infections among potential Blood donors in Ibadan, Nigeria. Afr J Clin Exp Microbiol.

[9] Apata IW, Averhoff F, Pitman J, Bjork A, Yu J, Amin NA (2014). Progress toward prevention of transfusion-transmitted hepatitis B and hepatitis C infection - sub-Saharan Africa, 2000-2011. MMWR Morb Mortal Wkly Rep.

[10] Avert Africa HIV and AIDS statistics 2011. http://www.avert.org/africa-hiv-aids-statistics.htm.

[11] Adindu A (2010). Assessing and assuring quality of health care in Africa. Afr J Med Sci.

[12] Oyeyemi SO, Gabarron E, Wynn R (2014). Ebola, Twitter, and misinformation: a dangerous combination?. BMJ.

